# Comparison of the novel WEst coast System for Triage (WEST) with Rapid Emergency Triage and Treatment System (RETTS©): an observational pilot study

**DOI:** 10.1186/s12245-022-00452-2

**Published:** 2022-09-12

**Authors:** Samah Habbouche, Tobias Carlson, Daniel Johansson, Schani Kjaerbeck, Mathias Malm, Per-Arne Svensson, Lina Holmqvist

**Affiliations:** 1 Gothenburg Emergency Medicine REsearch Group (GEMREG), Gothenburg, Sweden; 2 Emergency Department, Sahlgrenska University Hospital, Gothenburg, Sweden; 3 Emergency Development Center, Sahlgrenska University Hospital, Gothenburg, Sweden; 4 Institute of Medicine, Sahlgrenska Academy, University of Gothenburg, Gothenburg, Sweden; 5 Institute of Health and Care Science, Sahlgrenska Academy, University of Gothenburg, Gothenburg, Sweden

**Keywords:** Emergency triage system, Emergency department, Emergency triage level

## Abstract

**Background:**

Most Swedish emergency departments (ED) use the triage system Rapid Emergency Triage and Treatment System (RETTS©), which over time has proven to prioritize patients to higher triage levels. When many patients are prioritized to high triage levels, challenges with identifying true high-risk patients and increased waiting time for these patients has emerged. In order to achieve a more balanced triage in relation to actual medical risk, the triage system WEst coast System for Triage (WEST) was developed, based on the South African Triage Scale (SATS). The aim of this study was to perform an initial evaluation of the novel emergency triage system WEST compared to the existing RETTS©.

**Methods:**

Both RETTS© and WEST are five level triage systems illustrated by colors. Nurses from each of the three adult EDs of Sahlgrenska University Hospital in Gothenburg and the ambulance service assessed and triaged 1510 patients according to RETTS© and immediately thereafter filled out the WEST triage form. Data from each triage report were analyzed and grouped according to the triage color, chief complaint, and outcome of each patient. Data on discharge categories and events within 72 h were also collected. Data were analyzed with descriptive statistical methods.

**Results:**

In general, WEST displayed lower levels of prioritization compared to RETTS©, with no observed impact on patients’ medical outcomes. In RETTS© orange triage level, approximately 50% of the patients were down prioritized in WEST to yellow or green triage levels. Also, in the RETTS© yellow triage level, more than 55% were down prioritized to green triage level in WEST. The number of patients who experienced a serious event during the first 72 h was few. Three patients died, these were all prioritized to red triage level in RETTS©. In WEST two of these patients were prioritized to red triage level and one to orange triage level. All these patients were admitted to hospital before deterioration.

**Conclusions:**

WEST may reduce over prioritization at the ED, especially in the orange and yellow triage levels of RETTS©, with no observed increase in medical risk. WEST can be recommended for a clinical comparative study.

**Supplementary Information:**

The online version contains supplementary material available at 10.1186/s12245-022-00452-2.

## Background

Emergency triage is the systematic prioritization of patients according to their level of medical urgency [1]. Patients with the highest prioritization level will receive medical care immediately [2]. Despite a relative abundance of studies on different emergency triage systems, there is still no consensus as to which triage system has the best ability to discriminate urgencies from non-urgencies [3, 4]. This is probably due to differences in the structure of the triage systems and the methodology of the studies [3, 5, 6]. Several triage systems evaluate the patient's chief complaint in combination with vital parameters (VPs) using a standardized flow chart to make safe, fast, accurate, and reproducible prioritizations [4].

The most widely used triage system in Sweden is the Rapid Emergency Triage and Treatment system (RETTS©), previously named Medical Emergency Triage and Treatment system (METTS) which was developed from the Manchester Triage Scale (MTS) [7]. METTS/RETTS© is a five-level triage (red-orange-yellow-green-blue) system, where classification of triage level is made from an assessment of the chief complaint through an “Emergency Symptoms and Signs” (ESS)-protocol, together with VPs [8]. A previous study on RETTS© discriminatory ability showed positive results [8], but there are conflicting results regarding the reliability of its application by nurses [9, 10].

It has been observed that, over time, an increasing proportion of patients has been triaged to the second highest triage level in RETTS© (orange) at Sahlgrenska University Hospital in Gothenburg, Sweden, reaching 20–27% in 2018 compared to the numbers from previous reports showing 14% of patients in that triage category [11]. When many patients are prioritized to high triage levels, challenges with identifying true high-risk patients and increased waiting time for these patients has emerged [12]. Furthermore, accumulation of patients with high triage levels is a component of ED crowding and thus has negative effects on the working environment of the emergency staff [13].

In 2018, a health technology assessment report concluded that triage systems based on the South African Triage Scale (SATS) may increase correct prioritization [3, 14, 15]. To achieve a more balanced triage in relation to the actual medical risk, a triage system called the WEst coast System for Triage (WEST) was developed. The aim of this study is to perform an initial evaluation of the novel emergency triage system WEST compared with the existing RETTS©. We hypothesized that WEST would reduce the number of patients in the second highest triage level (orange).

## Methods

### Development of WEST

**Fig. 1:**
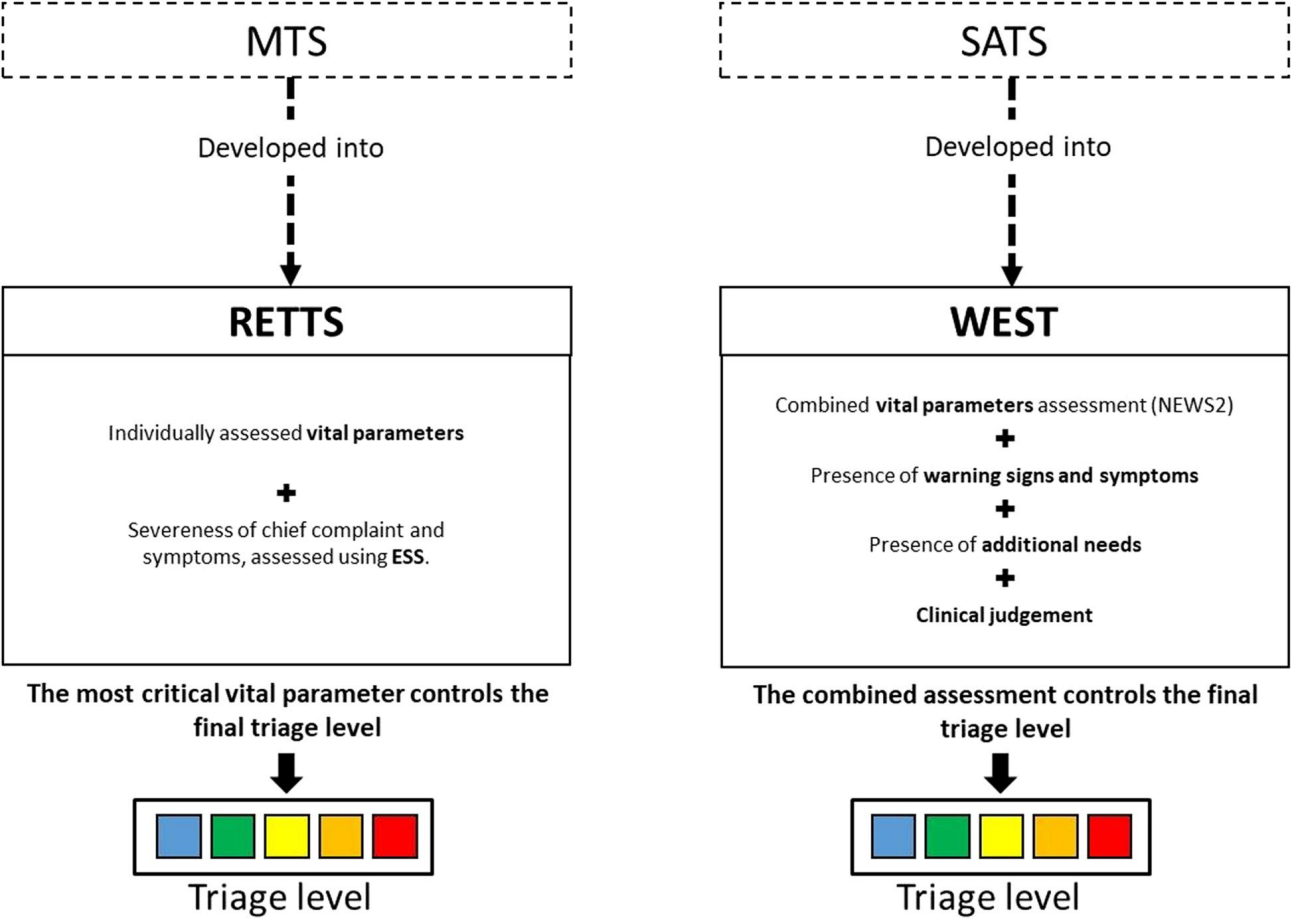
Development and components of the different triage systems. ESS = Emergency Symptoms and Signs, NEWS2 = National Early Warning Score2, additional needs, for example, frailty or a need for direct hospitalization

South African Triage Scale (SATS) is a five-level triage (red-orange-yellow-green-blue) system, where classification of triage level is made from assessment of clinical signs, VPs and clinical judgement of emergency care staff [16]. SATS guides the staff to look for clinical signs and symptoms that directly classify the patient into one out of three categories: emergency (red), very urgent (orange), or urgent (yellow). With SATS, SATS Norway, and SATS at St. Goran Hospital in Sweden as a model, the staff at the Emergency Development Center (EDC) at Sahlgrenska University Hospital developed WEST (Fig. 1) in close co-operation with specialists representing different fields of medicine. With nominal group technique, the group agreed on how to perform risk assessment, according to clinical signs and symptoms named “warning signs and symptoms.” According to the description of the warning signs and symptoms, the patient can be directly categorized to their triage severity level or, if the chief complaint does not correspond to a specific warning sign and symptom, the patient will be prioritized according to other variables such as the VPs and the clinical judgement by the triaging staff. As in the original SATS, WEST uses a calculated score for assessment of VPs [17]. With an accumulated score of VPs deterioration is easily detected. In WEST, surveillance of VPs performed using the updated version of the National Early Warning Score (NEWS2) [18]. The score harmonizes with in-hospital care at Sahlgrenska University Hospital where NEWS2 is already implemented. One ambition with WEST was to utilize the competence of the ED care staff and thus the developers of WEST decided that the clinical judgement should be an outspoken variable in the triage system enabling the triaging team to use clinical judgement in their final prioritization [19]. The clinical judgement is an overall clinical assessment by the triaging nurse, based on how the patient looks and acts, the so-called clinical gestalt. In addition, the clinical judgement considers the patients previous health history and condition. For example, a cold and diaphoretic patient with chest pain, previous heart disease with normal ECG finding, green NEWS2 and yellow warnings sign can be upgraded to the orange (second highest) triage level. Since red and orange triage levels are indicators of high urgency, clinical judgement alone can never be used to down prioritize patients. Another potential feature of WEST is the possibility to include other elements of care, such as need for direct admission to hospital and frailty assessment in older patients [20]. Thus, WEST uses the combined assessment of warnings signs and symptoms, VPs, clinical judgement and additional needs (Fig. 1).

### Design, study population, and education of test nurses

This is an observational descriptive pilot study where nurses either in the ED or in the ambulance service triaged each patient with both RETTS© and WEST. Patients, both walk-ins and arriving by ambulance at the three adult EDs (ED 1–3) of Sahlgrenska University Hospital, Gothenburg Sweden, were included in the study. Patients where the ambulance bypassed the ED and admitted patients directly for inpatient care, children < 16 years of age transported to children’s hospital and patients triaged when none of the test nurses were on duty were excluded.

Six nurses, experienced in emergency triage, from each ED site and from the ambulance service (*N* = 24) were trained in how to perform an assessment according to WEST by the developers at the EDC. These nurses were defined as test nurses in the study. Education of test nurses was performed in small groups at each site for 4 h on 2 separate occasions. Test nurses were scheduled day, evening, and night shifts in the EDs and ambulance services. The prioritization, according to WEST, was made on triage forms immediately after the RETTS© prioritization, with the patient still in the triage room/ambulance. Patient assessment and triage in the ambulance service was conducted at scene with both RETTS© and WEST. Any changes of patients warning signs and symptoms or VPs during transport were documented but the final assessment priority at scene was included in the study.

### Data collection and statistical analysis

Calculations based on patient inflow indicated that a three-week sampling period would generate a study population of approximately 1500, which was deemed sufficient for a pilot study. No power calculation was performed. Data were continuously collected during a 3-week period (3 December–23 December 2018). Registration of WEST took only a few seconds-minutes to perform, and the instruction to the test nurses was to triage the patient as if WEST was the existing triage system. This was performed to minimize any bias by the existing triage system. The patients’ triage level during the ED visit was not changed but remained as if only the RETTS©-triage system was used.

All triage was performed using the Swedish version of the WEST flow sheet including warning signs and symptoms and a data collection chart. The flow sheet and chart are attached as additional files (Additional file 1).

All patients prioritized according to both triage systems were eligible for data analysis. Data were registered on mode of arrival, chief complaint, RETTS©-ESS-code, VPs according to RETTS© and RETTS© triage-level. Furthermore, data on WEST color/prioritization, warning signs and symptoms of WEST, NEWS2 VPs, and the final clinical judgement for WEST were registered.

In addition, we used follow-up data from the patient administrative systems (Elvis 5.3), a system that supports registration, scheduling, and logistics at the hospital. Data were collected for discharge categories (admission to ward, admission to intensive care unit (ICU), discharge to home, referral from the hospital to other units and left ED without seeing a doctor). Furthermore, we registered outcome data such as admission to the ICU within 72 h, unplanned revisit to an ED within 72 h, and deceased within 72 h. Data on deaths were requested from the National Board of Health and Welfare. Data were also collected from the patient medical records (Melior 220, Cerner Corporation, Kansas City, USA) with basic content for clinical documentations, prescription and administration of medications, referral and replies, letters, and certificates. Additional functions are x-ray and laboratory test results.

Data files were also grouped and analyzed descriptively, according to the different subgroups of triage levels. The complete dataset was de-identified.

The analysis of the data was performed by descriptive statistics. Statistical analysis of the RETTS© and WEST final triage level was performed using Excel (Microsoft, CA, USA).

## Results

### Study population

In total, 1510 patients were double triaged (RETTS© and WEST) during the three-week period at the three adult EDs of Sahlgrenska University Hospital and the ambulance service. Characteristics of the patients in the study population and the chief complaint are listed in Table 1. Neurological deficits include neurological symptoms of stroke, paresthesia’s and visual disorders such as amaurosis fugax. Infections includes symptoms of infections such as couch, runny nose, pain upon swallowing, gastroenteritis, local skin infections with or without body temperature > 38.5, or isolated body temperature > 38.5.

**Table 1:** Characteristics of patients in the study population and number of each chief complaint of ED-visits

	All EDs	ED1	ED2	ED3	AMB
**Total number (*N*)**	1510	551	569	318	72
**Age, years (median) (range)**	52(12^a^–100)	54(12^a^–100)	47(33–95)	51(21–93)	67(19–97)
**Sex (w/m) (*n*/*n*)**	762/748	289/262	282/287	158/160	33/39
**Chief complaint (*n*)**					
*Abdominal pain*	263	115	143	1	4
*Chest pain*	136	60	65	5	6
*Infection*	124	47	55	16	6
*Other*^b^	118	50	50	12	6
*Hand trauma*	89	2	5	82	0
*Dyspnea*	72	28	25	9	10
*Other trauma*	69	10	15	39	5
*Musculoskeletal/extremity pain*	66	22	25	19	0
*Unspecified disease*	65	35	22	3	5
*Foot trauma*	61	1	5	54	1
*Arrhythmia/palpitations*	52	24	21	2	5
*Urological symptoms*	48	34	11	2	1
*Head trauma*	41	20	13	3	5
*Arm trauma*	40	2	0	38	0
*Headache*	39	15	21	3	0
*Vertigo/dizziness*	38	19	13	1	5
*Neurological deficits*	38	18	14	5	1
*Back pain*	31	6	11	14	0
*Rectal symptoms*	20	4	16	0	0
*Ear nose or throat problem*	16	10	6	0	0
*Wound (cut or laceration)*	15	10	5	0	0
*Allergy*	15	3	10	2	0
*Eye symptoms*	10	0	10	0	0
*Seizure*	9	3	1	0	5
*GI-bleed*	9	4	2	0	3
*Fainting/syncope*	9	4	3	1	1
*Post-operational*	9	2	0	7	0
*Intoxication*	8	3	2	0	3

Emergency department (ED) 1–3 and ambulance service (AMB)

^a^The emergency departments receive patients above the age of 16, although Ear, Nose and Throat subdivision received all ages, hence, age under 16 years found in this study

^b^Other: grouped chief complaints with less than nine patients registered

The age range for the patients in the study was 12–100 years. The under-aged patients included were due to patients visiting the Ear, Nose, and Throat subdivision at ED1. The number of women and men in the study population was almost equal. There were no major numerical differences in sex distribution between the sites. ED1 and ED2 have similarities in their case distribution due to equalities in subspecialty resources. At ED3, we found an overrepresentation of injuries to extremities, which is explained by ED3 being the only receiving center for orthopedic trauma in the study. The three most common causes for ED visits during the three-week period of registration were abdominal pain, chest pain, and infections. During the entire registration period, 13 level 1 trauma patients were admitted to the EDs, but none of them were included in the study population. In addition, 136 cases of intoxication were registered to the EDs during the entire registration period, out of which eight patients were included in the study population (Table 1).

### Evaluation of RETTS© and WEST triage levels

**Fig. 2:**
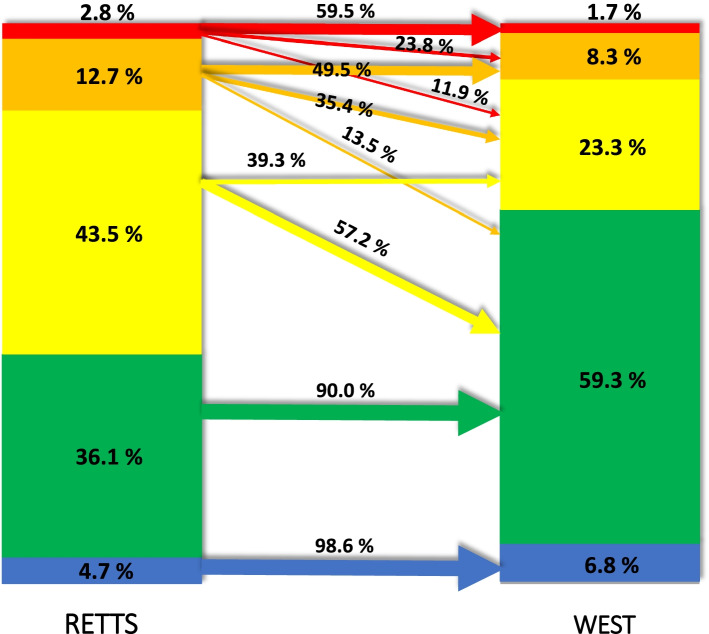
Distribution and comparisons between RETTS© and WEST prioritizing color. Data are presented as percentages for each prioritization color for both triage systems. Arrows indicate similarities (horizontal arrows) and differences (sloping arrows) in prioritization color between the two different triage systems (only arrows representing 10% or more are included in the figure)

Differences in priority levels between RETTS© and WEST are displayed in Fig. 2 and in the graphical abstract (Additional file 2). The most common triage level in RETTS© was yellow (43.5%), while most patients (59.3%) were triaged as green according to WEST. The main differences between the two triage systems were seen in RETTS© orange and yellow triage levels, seen as sloping arrows in Fig. 2. From the RETTS©-orange triage level, 49.5% of the patients were prioritized to lower triage levels with WEST. From this triage level, 35.4% were triaged to yellow and 13.5% to green. Furthermore, in the RETTS©-yellow triage level, 57.2% were prioritized to the lower triage level green with WEST. No major differences were seen in triage prioritization between the two triage systems in the green and the blue group (Fig. 2) (Additional file 2).

In 38 patients, the difference in prioritization differed by two triage levels or more (Table 2). The reason for the difference in the triage level in 12 of the patients was high heart rate or high respiratory rate that gave higher triage level in RETTS© compared to WEST (NEWS2). Two of the patients with red prioritization in RETTS© due to one single vital parameter had asymptomatic atrial fibrillation without any signs of decompensation. Other causes were found for twelve patients, and in this group, the symptom itself gave a higher prioritization in RETTS© compared to WEST. Four patients were prioritized orange in RETTS© due to use of anticoagulation with mild trauma or symptom of GI/urogenital bleeding, all these patients had a normal range in the vital parameters. Furthermore, two patients received a higher prioritization in RETTS© due to abnormal laboratory findings, such as high international normalized ratio or high creatinine level. Analysis of the medical journal revealed no serious outcome in the down prioritized patients in WEST.

**Table 2:** Patients where WEST triage level differed by two triage levels down or more compared to RETTS©

	Red to yellow	Orange to green	Orange to blue	Yellow to blue
**Cause**				
Single high vital parameter	3	8	1	0
Anticoagulation use and mild trauma or GI/URO-bleed	0	4	0	0
Neurological deficit	0	4	0	0
Laboratory	0	2	0	0
Other^a^	2	6	0	6

^a^Sudden headache, chest pain, melena, syncope, acute scrotum, pain, alcohol intoxication + mild trauma, wound, residual urine, 2 missing

### Evaluation of discharge categories and events within 72 h

Discharge categories are shown in Table 3 and events within 72 h in Table 4.

**Table 3:** RETTS© and WEST prioritization colors stratified by discharge category

RETTS©	WEST^a^	Discharge category				
		Home	Referral^b^	Ward	ICU	LWBS^c^
Red, 42	Red, 25	1(4%)	2(8%)	21(84%)	1(4%)	0
	Orange, 10	1(10%)	1(10%)	8(80%)	0	0
	Yellow, 5	2(40%)	0	3(60%)	0	0
	Green, 1	1(100%)	0	0	0	0
	Blue, 0	0	0	0	0	0
Orange, 192	Red, 0	0	0	0	0	0
	Orange, 95	47(49%)	3(3.1%)	43(45%)	1(1%)	1(1%)
	Yellow, 68	43(63%)	1(1.5%)	23(34%)	0	1(1.5%)
	Green, 26	16(61.5%)	2(7.7%)	8(31%)	0	0
	Blue, 1	0	1(100%)	0	0	0
Yellow, 657	Red, 1	1(100%)	0	0	0	0
	Orange, 13	6(46%)	0	7(54%)	0	0
	Yellow, 258	181(70%)	26(10%)	39(15%)	0	12(4.6%)
	Green, 376	265(70%)	54(14%)	37(10%)	0	20(5%)
	Blue, 6	5(83%)	0	0	0	1(17%)
Green, 546	Red, 0	0	0	0	0	0
	Orange, 7	3(43%)	0	4(57%)	0	0
	Yellow, 22	17(77%)	1(4.5%)	4(18%)	0	0
	Green, 491	283(58%)	173(35%)	15(3%)	0	20(4%)
	Blue, 26	17(65%)	6(23%)	1(4%)	0	2(8%)
Blue, 71	Red, 0	0	0	0	0	0
	Orange, 0	0	0	0	0	0
	Yellow, 0	0	0	0	0	0
	Green, 1	0	1(100%)	0	0	0
	Blue, 70	34(48.6%)	33(47%)	2(3%)	0	1(1.4%)

^a^Prioritization color missing for 6 WEST registrations (1 red, 2 orange, 3 yellow)

^b^Patients referred from the ED to primary care centers or other health care centers

^c^LWBS, left without being seen

**Table 4:** RETTS© and WEST prioritization color stratified by events within 72 h

RETTS©	WEST*	Events within 72 h			
		Planned revisit	Unplanned revisits	Death	ICU
Red, 42	Red, 25	0	0	2 (8%)	0
	Orange, 10	0	0	1(10%)	0
	Yellow, 5	0	0	0	0
	Green, 1	0	0	0	0
	Blue, 0	0	0	0	0
Orange, 192	Red, 0	0	0	0	0
	Orange, 95	1(1%)	6(6.3%)	0	1(1%)
	Yellow, 68	1(1.5%)	5(7.3%)	0	0
	Green, 26	1(3.8%)	2(7.7%)	0	0
	Blue, 1	0	0	0	0
Yellow, 657	Red, 1	0	0	0	0
	Orange, 13	0	0	0	0
	Yellow, 258	7(2.7%)	13(5%)	0	0
	Green, 376	8(2.1%)	18(4.8%)	0	0
	Blue, 6	0	1(16.6%)	0	0
Green, 546	Red, 0	0	0	0	0
	Orange, 7	0	0	0	0
	Yellow, 22	0	3(13.6%)	0	0
	Green, 491	8(1.6%)	29(6%)	0	0
	Blue, 26	0	1(3.8%)	0	0
Blue, 71	Red, 0	0	0	0	0
	Orange, 0	0	0	0	0
	Yellow, 0	0	0	0	0
	Green, 1	0	0	0	0
	Blue, 70	1(1.4%)	5(7%)	0	0

Out of the total study population (*N* = 1510), 215 patients were admitted to the ward, which accounts for about 14% of the study population (Table 3). Two patients (0.13%) out of 1510 patients were admitted to the ICU directly (Table 3). Both patients had the same priority in WEST and RETTS© (one patient red and one patient orange).

Three patients died within 72 h from the time of arrival to the ED. All these patients were triaged as red according to RETTS©; however, one was triaged as orange in WEST (Table 4). All these patients were admitted to hospital prior to their death. One patient, orange triage level in both RETTS© and WEST, was admitted to ICU within 72 h (Table 4). This patient was admitted to a ward prior to deterioration. In general, unplanned re-visits within 72 h from the ED visit in each group reached the level of 5–8% (Table 4).

## Discussion

In general, WEST displayed lower levels of prioritization compared to RETTS©. Down prioritization with WEST was seen in approximately half of the patients in the RETTS© orange and yellow triage levels. Hence, use of WEST may reduce over prioritization in the ED compared with the use of RETTS©. No major safety issues were observed with WEST compared with RETTS©.

With an increasing number of patients being prioritized to the second highest triage level, orange, it is challenging to find those patients who are in the greatest need of emergency care. In RETTS© orange triage level, 55% of patients were discharged home, compared to 45% in WEST orange triage level. This underlines the problem with RETTS© over triage. The use of a triage system like WEST may not only reduce over-prioritization in the ED, but it may also help focus ED resources on the patients that need urgent care and monitoring.

The comparisons of discharge pattern and events within 72 h in this study, using both RETTS© and WEST triage systems in all patients, do not reveal any major differences. Hence, the results open for further clinical evaluation of WEST.

One patient within the RETTS© red triage level was prioritized to orange triage level in WEST and died within 72 h. This patient had history of myocardial infarction, dementia, psychological illness, and diabetes. The patient was admitted due to myocardial infarction which was treated conservatively. Since this patient was stable upon admission and treated conservatively, an orange triage level at the ED was relevant. The patient deteriorated and died from pulmonary edema and heart failure on day three following admission. Upon admittance to medical ward an “do not resuscitate” order was taken before deterioration and death.

Direct comparison between the results from our study and other studies is challenging due to the heterogeneity of existing studies. However, discharge categories and events within 72 h were lower in our study compared to a study by Gräff et al. [21], with admission to the ward (14% vs. 27%), admission to the ICU (0.07% vs. 5.1%), and in hospital mortality (0.2% vs. 1.3%), respectively. This comparison indicates that seriously ill patients are underrepresented in the present study and underlines the need for further evaluation of the safety of WEST in the whole range of ED patients.

### Strengths and limitations

The main strength of this study is the number of study patients, which is relatively large for a pilot study of a triage system. Another strength is the wide range and robustness of parameters that were investigated. Finally, our study represents real-world data and has a high degree of relevance to many EDs.

The study has certain limitations that need to be acknowledged. First, the study is descriptive and there is no statistical comparison between the two triage systems. Secondly, the study aimed to test triage in a study population representative of all the patients arriving at the EDs. Consequently, the number of seriously ill patients is small, not only in the red triage level with highest priority but also in patients admitted directly to the ICU. This can be explained by the relatively low number of patients registered by the ambulance service, which is the main way of transportation of seriously ill patients to hospitals [19]. This also explains why there are no level 1 trauma patients described in the study population and relatively few intoxications [20]. Direct admissions to ICU and level 1 traumas were not excluded from this study rather we were not able to register them since most test-nurses at ED 1–3 were placed in the triage area and we had few registrations in total from the prehospital test-nurses. In both RETTS© and WEST, when a patient is critically ill, they get assigned and registered with the highest level of priority (red) directly, the ambulance alerts the ED before arriving and the hospital team gives emergency care directly to the patient. There was no retrospective triage, instead these patients bypassed the registration process of study participants. Therefore, we can conclude that patients prioritized to the highest triage level are underrepresented in this study. Thus, we cannot draw any conclusions about the differences between RETTS© and WEST at the highest priority level.

Furthermore, since test nurses in the ambulance only take care of one patient at a time compared to test nurses at the ED the triage assessment in the ED can differ from the triage assessment in the ambulance. Although this can be an important difference in the triage assessment this does not change the outcome of our study since we compare differences between two different triage systems and not the triage sites. Even though two separate triage forms for the two triage systems were used, test nurses may have been biased in their clinical assessment since RETTS© is the existing and well-known triage system. Generalizability is probably high for other EDs where RETTS© is used. However, generalizability is unclear for EDs where other triage systems are used.

## Conclusions

WEST may reduce over prioritization at the ED, especially in the orange and yellow triage levels of RETTS©, with no observed increase in medical risks. Thus, WEST can be recommended for a clinical comparative study.

## Supplementary Information

**Additional file 1.** WEST flow sheet with warning signs and symptoms in Swedish and data collection chart in English.

**Additional file 2.** Graphical abstract.

## Data Availability

The data that support the findings of this study are available from Sahlgrenska University Hospital but restrictions apply to the availability of these data, which were used under license for the current study, and so are not publicly available. Data are however available from the authors upon reasonable request and with extended approval from ethical review board.

## Abbreviations

EDC: Emergency Development Center
ED: Emergency departments
ESS: Emergency symptoms and signs
ICU: Intensive care unit
MTS: Manchester Triage Scale
METTS: Medical Emergency Triage and Treatment system
NEWS2: National Early Warning Score
RETTS©: Rapid Emergency Triage and Treatment System
SATS: South African Triage Scale
VP: Vital parameter
WEST: WEst coast System for Triage
