# Supplementary material for: Comparison of the novel WEst coast System for Triage (WEST) with Rapid Emergency Triage and Treatment System (RETTS©): an observational pilot study
Source: Int J Emerg Med. 2022 Sep 12;15:47. doi: 10.1186/s12245-022-00452-2 (PMC9465908; doi:10.1186/s12245-022-00452-2)
Supplement: Supplementary file 1 — Additional file 1. WEST flow sheet with warning signs and symptoms in Swedish and data collection chart in English. [file 12245_2022_452_MOESM1_ESM.pdf]

RÖD VARNINGSSYMTOM

BLÖDNING/ANEMI
- Blödning, stor och okontrollerad
- Hematemes / pågående melena med puls >120

BRÖSTSMÄRTA
- Bröstsmärta med ST-höjning/ nytillkommet grenblock
- Bröstsmärta med svimning eller pågående vegetativa symtom

BUK/RECT/SCROTUM/LJUMSKE
- Buksmärta, plötsligt påkommen, svår (nivå 3) och konstant med SBT< 90 och/eller puls >120

DIABETES
- Hypoglykemi Glc <3

DYSPNE
- Dyspné med gurglande, skummande andning
- Dyspné, kraftig med ST-höjning/ nytillkommet grenblock

GRAVIDITET / POST PARTUM
- Gravid >v12 med kraftig vaginalblödning (blödning genom 2 bindor/h i mer än 2h)
- Gravid med pågående eller genomgånget krampanfall (tom 2v efter födsel)
- Gravid >v20 med snabbt insättande starka buksmärtor
- Gravid >v20 med STB >180 eller DBT >120

HJÄRTSTOPP / LUFTVÄGSHINDER
- Hjärtstopp; pågående/åtgärdat
- Luftväg; stridor, hotad eller ofri luftväg,intub/larynxmask

INFEKTION / FEBER
- Infektion, misstänkt + NEWS2 ≥7
- Infektion med feber, påverkad och nackstelhet/ meningism/peteccier

MEDVETANDEPÅVERKAN
- Medvetslös

NEURO / STROKE
- Stroke med symtom <6h och modifNIHSS ≥2p
- Yrsel akut påkommen med symtom <6h och modifNIHSS ≥2p
- Kramper pågående

OSPECIFIK SJUKDOM
- Kalium >6,5 + EKG-förändringar

TRAUMA

Tramalarm Nivå 1

Andning
- Behov av ventilationsstöd
- AF < 10 eller > 29

Cirkulation
- BT < 90 eller ej palpabel radialispuls

Neurologi
- RLS ≥ 3 eller GCS ≤ 13*

Skademekanism
- Penetrerande våld mot huvud, hals, bål, extremiteter ovan armbåge/knä (inte ytliga skärsår)*
- Öppen skallskada/impressionsfraktur*
- Ansikts-/halsskada med hotad luftväg
- Instabil/deformerad bröstkorg*
- Svår smärta i bäckenet (misstänkt bäckenfraktur)*
- Misstänkt ryggmärgsskada*
- ≥ 2 frakturer på långa rörben
- Amputation ovan hand/fot*
- Stor yttre blödning
- Brännskada ≥ 18 % eller inhalationsskada*

TRAUMA

Tramalarm Nivå 2

Skademekanism
- Bilolycka > 50 km/h utan bilbälte
- Utkastad ur fordon
- Fastklämd med losstagning > 20 min
- MC-olycka (eller motsvarande) > 35 km/h
- Fall > 5 m

Tramalarm Nivå 3

Skademekanism
- Fall >3m
- Trafikolycka med misstanke om högenergivåld
- MC/Moped >30km/h
- Hästolyckor
- Oskyddade trafikanter

TRAUMA
- Buktrauma med tilltagande smärta/omfång, urostopp eller hematuri
- Drunkning/dykolycka
- Elolycka högspänning / blixt
- Fraktur med misstänkt kärlskada (nedsatt cirkulation distalt, blek och kall extremitet), nervskada
- Hängning/strypning
- Kemolycka / strålskada
- Ögonskada; penetrerande eller frätande

ORANGE VARNINGSSYMTOM

ALLERGI
- **Allergi**, akut och potentiellt allvarlig

BLÖDNING/ANEMI
- **Hematemes / pågående melena**
- **Bakre näsblödning / postop tonsillblödning**
- **Riklig vaginalblödning** (blödning genom 2 bindor/h i mer än 2h)

BRÖSTSMÄRTA
- **Bröstsmärta** med nytillkomna EKG-förändring
- **Bröstsmärta**, pågående med misstanke om AKS eller annan allvarlig orsak

BUK/RECT/SCROTUM/LJUMSKE
- **Buksmärta,** plötsligt påkommen och svår (nivå 3) eller vegetativa symtom
- **Scrotumsmärta**, hastigt påkommande, kraftig och ihållande

DIABETES
- **Hyperglykemi**, B-Glc >11 + AF ≥v22 eller metabol acidos

DYSPNE
- **Dyspne**, akut svår/synligt ansträngd andning/cyanos/utmattad patient
- **Dyspné,** nytillkommen med nytillkomna EKG-förändringar

EXTREMITETSPROBLEM
- **Akut ischemi** (blek och kall extremitet)

GRAVIDITET / POST PARTUM
- **Gravid** med något måttlig vaginalblödning (mer än kraftig mens)
- **Gravid** med kraftig buksmärta, ihållande eller intervallsmärta
- **Gravid** >v20 + huvudvärk / buksmärta / bröstsmärta/kräkning + BT >140/90 (preeklampsi)
- **Gravid** >v20 med trauma mot buk
- **Post-partum** (inom 14 d) med feber >38

HJÄRTRYTM
- **Tackycardi,** breddökad (som orsak till vårdtillfället)

HUVUDVÄRK
- **Huvudvärk**, urakut debut och intensiv

INFEKTION / FEBER
- **Infektion**, misstänkt samt NEWS2 5-6
- **Infektion**, misstänkt med pågående cytostatikabehandling / immunosuprimerad

INTOX
- **Intox,** akut och allvarlig eller suicidal

MEDVETANDEPÅVERKAN
- **Medvetandesänkt** nytillkommet

NEURO / STROKE
- **Stroke** med symtom 6-24h och modifNIHSS ≥2p
- **Yrsel** akut påkommen med symtom 6-24h och modifNIHSS ≥2p

OSPECIFIK SJUKDOM
- Känd addison eller binjurebarkssvikt

RYGGSMÄRTA
- **Ryggsmärta** med nytillkommen ridbyxanestesi eller inkontinens

SVIMNING
- **Svimning** helt utan förkänning

TRAUMA
- **Thoraxtrauma** med vegetativa symtom
- **Brännskada/frätskada 5-18% eller cirkumferent**
- **Fraktur** ; öppen, felställd, eller höftnära
- **Ledluxation (ej fingrar/tår), protesluxation eller luxationsfraktur**
- **Skalltrauma** med:
- GCS 14
- GCS 15 och 1 av: Blödningsrisk (Waran/NOAK, dubbel trombocythämning eller blödarsjuka) krampanfall, neurologiska avvikelser, shuntbehandling
- **Symtomgivande rökinhalation**

GUL VARNINGSSYMTOM

BLÖDNING/ANEMI
- **Blödning**; stor men kontrollerad
- **Anemi,** Hb <70 utan pågående blödning

BRÖSTSMÄRTA
- **Bröstsmärta**, pågående men med låg misstanke allvarlig orsak och utan tydlig palpömhet
- **Bröstsmärta** senaste 24h, ej pågående men med misstanke om AKS eller annan allvarlig orsak
- **Bröstsmärta,** andningskorrelerad med misstanke om allvarlig orsak

DIARRE/KRÄKNING
- **Kräkningar**, pågående

EXTREMITETSPROBLEM
- **Extremitetssmärta/-svullnad** med nytillkommen dyspné
- **Intraartikulär svullen, rodnad led** (en led) med feber/misstänkt infektion

NEURO / STROKE
- **TIA** senaste 24h – nu symtomfri

TRAUMA
- **Fraktur,** stark misstanke och aktuellt (72h) skadetillfälle
- **Ledluxation fingrar och tår**
- **Nackskada** med palpömhet i medellinjen
- **Skada** med oproportionerlig smärtnivå
- **Skalltrauma,** GCS 15; varit avsvimmad, amnesi eller upprepade kräkningar

URINVÄGSBESVÄR
- **Urostopp**

GUL BEHOV/PROCESS

SANNOLIKT SLUTENVÅRDSBEHOV
- **Sannolikt slutenvårdsbehov**

SKÖR ÄLDRE
- **Skör äldre** enl screening

YTTRE VÅLD
- **Sexuella övergrepp,** misstanke
- **Våld i nära relation,** misstanke

INGET VARNINGSSYMTOM FINNS

PATIENT-ID	ED1/ED2/ED3/AMB
	DATE/TIME:

**RR:**	
**SAT:**	
**BP:**	
**HR:**	
**Temp:**	
**RLS/GCS:**	
**Known resp-insuff.**	**YES**
**Oxygen**	**YES**

**Physiological parameter**	**Score** 3	2	1	0	1	2	3
Respiration rate (per minute)	≤8		9–11	12–20		21–24	≥25
SpO_2 Scale 1 (%)	≤91	92–93	94–95	≥96			
SpO_2 Scale 2 (%)	≤83	84–85	86–87	88–92<br>≥93 on air	93–94 on oxygen	95–96 on oxygen	≥97 on oxygen
Air or oxygen?		Oxygen		Air			
Systolic blood pressure (mmHg)	≤90	91–100	101–110	111–219			≥220
Pulse (per minute)	≤40		41–50	51–90	91–110	111–130	≥131
Consciousness				Alert			CVPU
Temperature (°C)	≤35.0		35.1–36.0	36.1–38.0	38.1–39.0	≥39.1	

RETTS chief complaint /ESS nr:

**RETTS ESS color**	RED	ORANGE	YELLOW	GREEN	BLUE
**RETTS prio**	RED	ORANGE	YELLOW	GREEN	BLUE

**WEST warning signs and symtom**	RED	ORANGE	YELLOW	
**Color according to NEWS 2 Medical risk**	**>7p High**	**5-6P Medium**	**single parameter 3p Low-medium**	0-4p Low

**Clinical judgement**	RED	ORANGE	YELLOW	GREEN	BLUE
**WEST prioritization**	RED	ORANGE	YELLOW	GREEN	BLUE

Comments:

Signature:
