# Supplementary material for: Comparison of the novel WEst coast System for Triage (WEST) with Rapid Emergency Triage and Treatment System (RETTS©): an observational pilot study
Source: Int J Emerg Med. 2022 Sep 12;15:47. doi: 10.1186/s12245-022-00452-2 (PMC9465908; doi:10.1186/s12245-022-00452-2)
Supplement: Supplementary file 2 — Additional file 2. Graphical abstract. [file 12245_2022_452_MOESM2_ESM.pdf]

Method:
1510 ED patient triaged with both triaging system

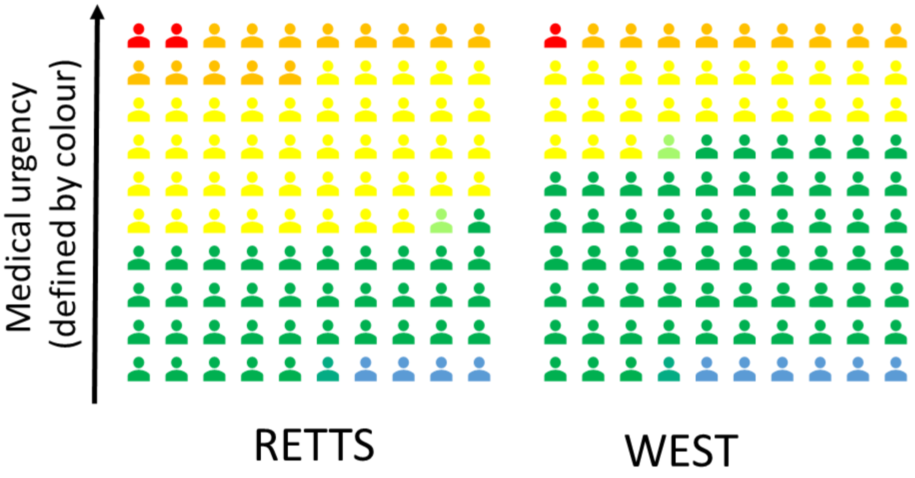

Results and conclusions

WEST may reduce overprioritization
